# {μ-[2-(Dimethyl­amino)phen­yl](2-fluoro­phen­yl)methano­lato}penta­methyl­dialuminum(III)

**DOI:** 10.1107/S1600536809012252

**Published:** 2009-04-25

**Authors:** Aihong Gao, Qing Su, Ying Mu

**Affiliations:** a State Key Laboratory of Supramolecular Structures and Materials, School of Chemistry, Jilin University, Changchun 130012, People’s Republic of China; b School of Chemistry, Jilin University, Changchun 130012, People’s Republic of China

## Abstract

Each of the Al atoms in the title compound, [Al_2_(CH_3_)_5_(C_15_H_15_FNO)], is four-coordinated in a distorted tetra­hedral geometry. The dimethyl­aluminium centre is bound by the N and the O atoms of the (2-dimethyl­amino­phen­yl)(2-fluoro­phen­yl)methano­late ligand. The second Al atom is bound by the methano­late O atom and by three methyl C atoms. The crystal studied was a racemic twin with a 0.4 (2):0.6 (2) domain ratio.

## Related literature

For organoaluminum complexes, see: Atwood & Harvey (2001 ▶); Dechy-Cabaret *et al.* (2004 ▶); Izod (2002 ▶); Linton *et al.* (2001 ▶); Liu *et al.* (2000 ▶); Ma *et al.* (2005 ▶); Nomura *et al.* (2005 ▶). For the synthesis of the ligand, see: Al-Masri *et al.* (2004*a* ▶). For a discussion of chirality in the ligand, see: Al-Masri *et al.* (2004*b* ▶).
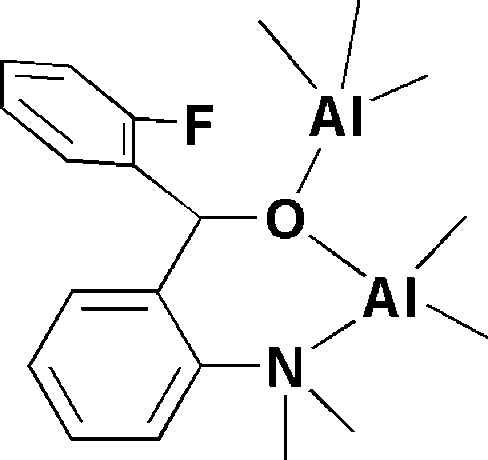


## Experimental

### 

#### Crystal data

[Al_2_(CH_3_)_5_(C_15_H_15_FNO)]
*M* *_r_* = 373.41
Orthorhombic, 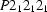

*a* = 9.1089 (7) Å
*b* = 13.1601 (10) Å
*c* = 18.3443 (15) Å
*V* = 2199.0 (3) Å^3^
*Z* = 4
Mo *K*α radiation
μ = 0.15 mm^−1^
*T* = 295 K
0.21 × 0.13 × 0.11 mm

#### Data collection

Bruker SMART CCD area-detector diffractometer
Absorption correction: multi-scan (*SADABS*; Bruker, 2001 ▶) *T*_min_ = 0.970, *T*_max_ = 0.984
12338 measured reflections
4318 independent reflections
3641 reflections with *I* > 2σ(*I*)
*R*_int_ = 0.039

#### Refinement

*R*[*F* ^2^ > 2σ(*F* ^2^)] = 0.055
*wR*(*F* ^2^) = 0.130
*S* = 1.09
4318 reflections
233 parameters
H-atom parameters constrained
Δρ_max_ = 0.65 e Å^−3^
Δρ_min_ = −0.17 e Å^−3^

### 

Data collection: *SMART* (Bruker, 1998 ▶); cell refinement: *SAINT* (Bruker, 1998 ▶); data reduction: *SAINT*; program(s) used to solve structure: *SHELXS97* (Sheldrick, 2008 ▶); program(s) used to refine structure: *SHELXL97* (Sheldrick, 2008 ▶); molecular graphics: *SHELXTL* (Sheldrick, 2008 ▶); software used to prepare material for publication: *SHELXTL*.

## Supplementary Material

Crystal structure: contains datablocks global, I. DOI: 10.1107/S1600536809012252/tk2388sup1.cif

Structure factors: contains datablocks I. DOI: 10.1107/S1600536809012252/tk2388Isup2.hkl

Additional supplementary materials:  crystallographic information; 3D view; checkCIF report

## Figures and Tables

**Fig. 1.**
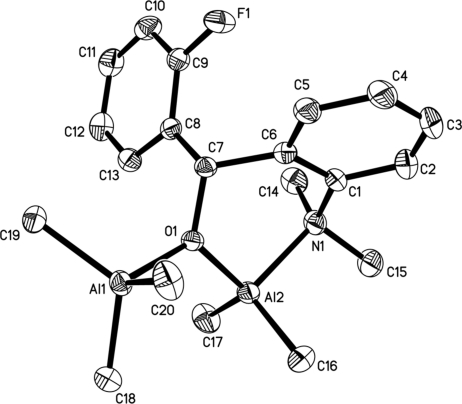
View of the molecule of (I) showing the atom labelling scheme. Displacement ellipsoids are drawn at the 30% probability level and H atoms have been omitted for reasons of clarity.

**Fig. 2.**
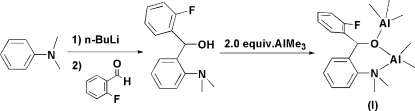
The formation of the title compound.

## supplementary crystallographic information

### Comment

Organoaluminum complexes have attracted considerable attention due to their rich structural chemistry (Izod, 2002), applications in organic synthesis (Linton *et al.*, 2001) and in catalytic chemistry (Dechy-Cabaret *et al.*, 2004; Liu *et al.*, 2000). A large number of ligands have been used to stabilize organoaluminum complexes and to tune the properties of their complexes. Among them, salen (Atwood & Harvey, 2001), salicylaldimine (Nomura *et al.*, 2005) and 1,4-dithiabutanediylbis(6-tert-butyl-4-methylphenol) ligands (Ma *et al.*, 2005) have attracted considerable attention. The aminophenylalcohol ligand, with a similar framework to the salicylaldimine ligand has been less well investigated. Herein, the structure of the title aluminium complex, (I), with the aminophenylalcohol ligand, is reported.

In the molecule of (I), Fig. 1, the Al atoms exist in different coordination environments, but with both adopting distorted tetrahedral geometries. The tetrahedral coordination around the Al1 atom involves three methyl-C atoms and the O1 atom from the deprotonated aminophenylalcohol ligand. The tetrahedral coordination around the Al2 atom involves the N1 atom and the O1 atom from the ligand and two methyl-C atoms. The (2-dimethylamino-phenyl)(2-fluoro-phenyl)methanolate ligand is therefore tridentate, bridging the two Al atoms via the O1 atom. The Al—Al separation within the dimer is 3.2631 (12) Å. The Al2—O distance (1.8165 (19) Å) is significantly shorter than the Al1—O distance (1.9199 (19) Å) because the former bond has more covalent chartacter. The six-membered chelate ring, O1/Al2/N1/C1/C6/C7, has a boat conformation with the C7 and N1 atoms occupying the apex positions. The dihedral angle between the two phenyl rings is 79.3°. Compound (I) was refined as a racemic twin.

### Experimental

The precursor compound (2-dimethylamino-phenyl)-(2-fluoro-phenyl)methanol was synthesized according to a modified literature procedure (Al-Masri *et al.*, 2004a). After removal of the solvent of a solution of nBuLi (79 ml, 79 mmol), *N*,*N*-dimethylaniline (10.0 ml, 79 mmol) was added at 0 °C with stirring, and the solution was slowly heated to 80 °C for 24 h, during which a yellow solid was formed. 2-FC_6_H_4_CHO (10.0 g, 79 mmol) in 40 ml of Et_2_O was added to the mixture at 0 °C. After stirring for 12 h, the reaction was quenched with H_2_O (30 ml), and the organic phase was separated, washed with brine, and dried over magnesium sulfate. The solvent was removed *in vacuo* to give the crude product as a yellow solid. The pure product was obtained by recrystallization from methanol as a white solid (13.9 g, 82%). The absolute configuration of the ligand could not be determined (Al-Masri *et al.*, 2004b). AlMe_3_ (4.0 ml, 1.0 *M* in toluene, 4.0 mmol) was added to a solution of (2-dimethylamino-phenyl)-(2-fluoro-phenyl)-methanol (0.49 g, 2.0 mmol) in toluene (20 ml) at -10 °C with stirring. The solution was gently heated to 60 °C for 24 h. After removal of the solvent, the product was crystallized from hexane and the desired complex (I) as a yellow crystalline solid (0.64 g, 87%) was obtained.

### Refinement

The C-bound H atoms were positioned geometrically with C—H = 0.93 (aromatic), 0.98 (methine) and 0.96 (methyl) Å, and allowed to ride on their parent atoms in the riding model approximation with *U*_iso_(H) = 1.2 (1.5 for methyl) *U*_eq_(C). The crystal studied was a racemic twin with a 0.4 (2):0.6 (2) domain ratio.

### Crystal data

**Table:**

[Al_2_(CH_3_)_5_(C_15_H_15_FNO)]	*D*_x_ = 1.128 Mg m^−^^3^
*M**_r_* = 373.41	Melting point: not measured K
Orthorhombic, *P*2_1_2_1_2_1_	Mo *K*α radiation, λ = 0.71073 Å
Hall symbol: P 2ac 2ab	Cell parameters from 4318 reflections
*a* = 9.1089 (7) Å	θ = 1.9–26.4°
*b* = 13.1601 (10) Å	µ = 0.15 mm^−^^1^
*c* = 18.3443 (15) Å	*T* = 295 K
*V* = 2199.0 (3) Å^3^	Block, yellow
*Z* = 4	0.21 × 0.13 × 0.11 mm
*F*(000) = 800	

### Data collection

**Table:**

Bruker SMART CCD area-detector diffractometer	4318 independent reflections
Radiation source: fine-focus sealed tube	3641 reflections with *I* > 2σ(*I*)
graphite	*R*_int_ = 0.039
Detector resolution: 9.00 cm pixels mm^-1^	θ_max_ = 26.0°, θ_min_ = 1.9°
φ and ω scans	*h* = −9→11
Absorption correction: multi-scan (*SADABS*; Bruker, 2001)	*k* = −16→13
*T*_min_ = 0.970, *T*_max_ = 0.984	*l* = −22→22
12338 measured reflections	

### Refinement

**Table:**

Refinement on *F*^2^	Primary atom site location: structure-invariant direct methods
Least-squares matrix: full	Secondary atom site location: difference Fourier map
*R*[*F*^2^ > 2σ(*F*^2^)] = 0.055	Hydrogen site location: inferred from neighbouring sites
*wR*(*F*^2^) = 0.130	H-atom parameters constrained
*S* = 1.09	*w* = 1/[σ^2^(*F*_o_^2^) + (0.0726*P*)^2^] where *P* = (*F*_o_^2^ + 2*F*_c_^2^)/3
4318 reflections	(Δ/σ)_max_ < 0.001
233 parameters	Δρ_max_ = 0.65 e Å^−^^3^
0 restraints	Δρ_min_ = −0.17 e Å^−^^3^

### Special details

**Table:**

Geometry. All esds (except the esd in the dihedral angle between two l.s. planes) are estimated using the full covariance matrix. The cell esds are taken into account individually in the estimation of esds in distances, angles and torsion angles; correlations between esds in cell parameters are only used when they are defined by crystal symmetry. An approximate (isotropic) treatment of cell esds is used for estimating esds involving l.s. planes.
Refinement. Refinement of F^2^ against ALL reflections. The weighted R-factor wR and goodness of fit S are based on F^2^, conventional R-factors R are based on F, with F set to zero for negative F^2^. The threshold expression of F^2^ > 2sigma(F^2^) is used only for calculating R-factors(gt) etc. and is not relevant to the choice of reflections for refinement. R-factors based on F^2^ are statistically about twice as large as those based on F, and R- factors based on ALL data will be even larger.

### Fractional atomic coordinates and isotropic or equivalent isotropic displacement parameters (Å^2^)

**Table:**

	*x*	*y*	*z*	*U*_iso_*/*U*_eq_	
Al1	1.10860 (9)	0.46062 (7)	0.20698 (4)	0.0355 (2)	
Al2	0.88654 (11)	0.37629 (6)	0.08119 (5)	0.0394 (2)	
C1	0.7128 (3)	0.5518 (2)	0.04877 (15)	0.0376 (6)	
C2	0.6729 (4)	0.6132 (3)	−0.00967 (18)	0.0564 (9)	
H2	0.6312	0.5839	−0.0509	0.068*	
C3	0.6939 (5)	0.7156 (3)	−0.00766 (19)	0.0622 (11)	
H3	0.6659	0.7555	−0.0471	0.075*	
C4	0.7561 (5)	0.7600 (3)	0.05242 (19)	0.0570 (10)	
H4	0.7724	0.8298	0.0535	0.068*	
C5	0.7942 (4)	0.7009 (2)	0.11100 (17)	0.0433 (7)	
H5	0.8360	0.7315	0.1517	0.052*	
C6	0.7722 (3)	0.5967 (2)	0.11129 (15)	0.0349 (6)	
C7	0.8237 (3)	0.5385 (2)	0.17944 (14)	0.0312 (6)	
H7	0.8830	0.5865	0.2077	0.037*	
C8	0.7058 (3)	0.4993 (2)	0.23137 (14)	0.0326 (6)	
C9	0.5929 (3)	0.5616 (2)	0.25606 (15)	0.0376 (7)	
C10	0.4867 (3)	0.5293 (3)	0.30503 (17)	0.0490 (8)	
H10	0.4127	0.5732	0.3200	0.059*	
C11	0.4926 (4)	0.4320 (3)	0.33089 (18)	0.0519 (9)	
H11	0.4211	0.4092	0.3631	0.062*	
C12	0.6030 (4)	0.3679 (3)	0.30967 (17)	0.0492 (8)	
H12	0.6075	0.3022	0.3281	0.059*	
C13	0.7082 (3)	0.4015 (2)	0.26058 (16)	0.0391 (7)	
H13	0.7827	0.3573	0.2467	0.047*	
C14	0.5573 (4)	0.4087 (3)	0.0817 (2)	0.0515 (8)	
H14A	0.5575	0.4322	0.1312	0.077*	
H14B	0.5498	0.3359	0.0810	0.077*	
H14C	0.4752	0.4376	0.0563	0.077*	
C15	0.6867 (5)	0.4024 (3)	−0.03245 (18)	0.0603 (10)	
H15A	0.5921	0.4194	−0.0520	0.090*	
H15B	0.6991	0.3299	−0.0330	0.090*	
H15C	0.7620	0.4334	−0.0615	0.090*	
C16	1.0316 (4)	0.4098 (3)	0.00634 (19)	0.0651 (11)	
H16A	1.0177	0.4788	−0.0093	0.098*	
H16B	1.0197	0.3650	−0.0345	0.098*	
H16C	1.1286	0.4021	0.0261	0.098*	
C17	0.8374 (5)	0.2368 (2)	0.1079 (2)	0.0615 (10)	
H17A	0.9130	0.2096	0.1387	0.092*	
H17B	0.8296	0.1962	0.0646	0.092*	
H17C	0.7455	0.2360	0.1334	0.092*	
C18	1.1997 (4)	0.3299 (3)	0.17878 (19)	0.0558 (9)	
H18A	1.1376	0.2749	0.1938	0.084*	
H18B	1.2937	0.3236	0.2020	0.084*	
H18C	1.2121	0.3279	0.1268	0.084*	
C19	1.0612 (3)	0.4677 (3)	0.31239 (15)	0.0426 (7)	
H19A	0.9969	0.5243	0.3212	0.064*	
H19B	1.1500	0.4764	0.3398	0.064*	
H19C	1.0136	0.4060	0.3272	0.064*	
C20	1.1980 (4)	0.5841 (3)	0.1655 (2)	0.0576 (10)	
H20A	1.1874	0.5833	0.1134	0.086*	
H20B	1.3004	0.5862	0.1779	0.086*	
H20C	1.1498	0.6430	0.1850	0.086*	
F1	0.5848 (2)	0.65789 (13)	0.23063 (10)	0.0526 (5)	
N1	0.6977 (3)	0.4407 (2)	0.04485 (13)	0.0419 (6)	
O1	0.92059 (19)	0.45697 (14)	0.15974 (9)	0.0319 (4)	

### Atomic displacement parameters (Å^2^)

**Table:**

	*U*^11^	*U*^22^	*U*^33^	*U*^12^	*U*^13^	*U*^23^
Al1	0.0302 (4)	0.0426 (5)	0.0336 (4)	0.0007 (4)	−0.0021 (4)	0.0045 (3)
Al2	0.0461 (5)	0.0358 (5)	0.0363 (4)	0.0089 (4)	−0.0048 (4)	−0.0070 (3)
C1	0.0364 (15)	0.0405 (17)	0.0358 (14)	0.0091 (14)	−0.0039 (12)	0.0024 (12)
C2	0.064 (2)	0.064 (2)	0.0409 (17)	0.013 (2)	−0.0107 (16)	0.0046 (15)
C3	0.086 (3)	0.052 (2)	0.049 (2)	0.018 (2)	−0.004 (2)	0.0183 (17)
C4	0.078 (3)	0.0336 (18)	0.059 (2)	0.0099 (18)	0.0115 (19)	0.0106 (15)
C5	0.0482 (19)	0.0369 (17)	0.0449 (16)	0.0039 (15)	0.0052 (15)	0.0023 (13)
C6	0.0342 (15)	0.0350 (16)	0.0355 (14)	0.0051 (13)	0.0041 (12)	0.0003 (12)
C7	0.0328 (14)	0.0279 (13)	0.0331 (13)	0.0020 (12)	−0.0032 (11)	−0.0063 (11)
C8	0.0262 (15)	0.0406 (15)	0.0311 (13)	0.0002 (12)	−0.0038 (11)	−0.0040 (11)
C9	0.0395 (17)	0.0361 (16)	0.0371 (14)	0.0012 (14)	−0.0048 (14)	−0.0040 (12)
C10	0.0313 (16)	0.067 (2)	0.0489 (18)	0.0017 (16)	0.0049 (14)	−0.0135 (17)
C11	0.0396 (18)	0.069 (2)	0.0469 (18)	−0.0107 (17)	0.0071 (15)	0.0043 (17)
C12	0.052 (2)	0.0467 (18)	0.0488 (18)	−0.0114 (17)	−0.0033 (16)	0.0061 (13)
C13	0.0352 (16)	0.0389 (17)	0.0433 (16)	−0.0003 (13)	−0.0017 (13)	−0.0010 (13)
C14	0.0427 (18)	0.051 (2)	0.061 (2)	−0.0049 (15)	−0.0136 (16)	−0.0063 (16)
C15	0.071 (3)	0.066 (2)	0.0437 (18)	0.009 (2)	−0.0214 (18)	−0.0159 (16)
C16	0.063 (2)	0.093 (3)	0.0397 (18)	0.023 (2)	0.0042 (17)	−0.0035 (19)
C17	0.074 (3)	0.0376 (19)	0.073 (2)	0.0052 (17)	−0.020 (2)	−0.0111 (16)
C18	0.049 (2)	0.068 (2)	0.0507 (19)	0.0196 (18)	−0.0025 (16)	0.0013 (17)
C19	0.0427 (17)	0.0503 (19)	0.0349 (15)	0.0009 (15)	−0.0037 (12)	−0.0006 (13)
C20	0.047 (2)	0.062 (2)	0.063 (2)	−0.0124 (18)	−0.0082 (17)	0.0220 (18)
F1	0.0529 (12)	0.0404 (10)	0.0646 (11)	0.0095 (8)	0.0089 (10)	−0.0038 (8)
N1	0.0441 (15)	0.0428 (15)	0.0387 (13)	0.0033 (12)	−0.0117 (12)	−0.0068 (11)
O1	0.0298 (10)	0.0329 (10)	0.0331 (9)	0.0040 (8)	−0.0009 (7)	−0.0037 (7)

### Geometric parameters (Å, °)

**Table:**

Al1—O1	1.9200 (19)	C11—C12	1.369 (5)
Al1—C20	1.971 (3)	C11—H11	0.9300
Al1—C18	1.978 (3)	C12—C13	1.387 (4)
Al1—C19	1.984 (3)	C12—H12	0.9300
Al2—O1	1.8165 (19)	C13—H13	0.9300
Al2—C17	1.952 (4)	C14—N1	1.506 (4)
Al2—C16	1.956 (4)	C14—H14A	0.9600
Al2—N1	2.030 (3)	C14—H14B	0.9600
C1—C2	1.391 (4)	C14—H14C	0.9600
C1—C6	1.399 (4)	C15—N1	1.508 (4)
C1—N1	1.471 (4)	C15—H15A	0.9600
C2—C3	1.361 (5)	C15—H15B	0.9600
C2—H2	0.9300	C15—H15C	0.9600
C3—C4	1.370 (5)	C16—H16A	0.9600
C3—H3	0.9300	C16—H16B	0.9600
C4—C5	1.372 (4)	C16—H16C	0.9600
C4—H4	0.9300	C17—H17A	0.9600
C5—C6	1.386 (4)	C17—H17B	0.9600
C5—H5	0.9300	C17—H17C	0.9600
C6—C7	1.540 (4)	C18—H18A	0.9600
C7—O1	1.435 (3)	C18—H18B	0.9600
C7—C8	1.525 (4)	C18—H18C	0.9600
C7—H7	0.9800	C19—H19A	0.9600
C8—C9	1.391 (4)	C19—H19B	0.9600
C8—C13	1.395 (4)	C19—H19C	0.9600
C9—F1	1.352 (3)	C20—H20A	0.9600
C9—C10	1.387 (4)	C20—H20B	0.9600
C10—C11	1.366 (5)	C20—H20C	0.9600
C10—H10	0.9300		
			
O1—Al1—C20	102.42 (12)	C8—C13—H13	118.9
O1—Al1—C18	103.56 (13)	N1—C14—H14A	109.5
C20—Al1—C18	116.26 (16)	N1—C14—H14B	109.5
O1—Al1—C19	104.29 (11)	H14A—C14—H14B	109.5
C20—Al1—C19	115.31 (16)	N1—C14—H14C	109.5
C18—Al1—C19	112.79 (14)	H14A—C14—H14C	109.5
O1—Al2—C17	112.96 (14)	H14B—C14—H14C	109.5
O1—Al2—C16	108.04 (14)	N1—C15—H15A	109.5
C17—Al2—C16	122.89 (17)	N1—C15—H15B	109.5
O1—Al2—N1	99.28 (10)	H15A—C15—H15B	109.5
C17—Al2—N1	106.28 (15)	N1—C15—H15C	109.5
C16—Al2—N1	104.34 (14)	H15A—C15—H15C	109.5
C2—C1—C6	119.1 (3)	H15B—C15—H15C	109.5
C2—C1—N1	121.1 (3)	Al2—C16—H16A	109.5
C6—C1—N1	119.8 (2)	Al2—C16—H16B	109.5
C3—C2—C1	121.2 (3)	H16A—C16—H16B	109.5
C3—C2—H2	119.4	Al2—C16—H16C	109.5
C1—C2—H2	119.4	H16A—C16—H16C	109.5
C2—C3—C4	120.2 (3)	H16B—C16—H16C	109.5
C2—C3—H3	119.9	Al2—C17—H17A	109.5
C4—C3—H3	119.9	Al2—C17—H17B	109.5
C3—C4—C5	119.5 (3)	H17A—C17—H17B	109.5
C3—C4—H4	120.2	Al2—C17—H17C	109.5
C5—C4—H4	120.2	H17A—C17—H17C	109.5
C4—C5—C6	121.9 (3)	H17B—C17—H17C	109.5
C4—C5—H5	119.1	Al1—C18—H18A	109.5
C6—C5—H5	119.1	Al1—C18—H18B	109.5
C5—C6—C1	118.1 (3)	H18A—C18—H18B	109.5
C5—C6—C7	116.8 (3)	Al1—C18—H18C	109.5
C1—C6—C7	125.0 (2)	H18A—C18—H18C	109.5
O1—C7—C8	109.7 (2)	H18B—C18—H18C	109.5
O1—C7—C6	110.8 (2)	Al1—C19—H19A	109.5
C8—C7—C6	117.5 (2)	Al1—C19—H19B	109.5
O1—C7—H7	106.0	H19A—C19—H19B	109.5
C8—C7—H7	106.0	Al1—C19—H19C	109.5
C6—C7—H7	106.0	H19A—C19—H19C	109.5
C9—C8—C13	115.5 (3)	H19B—C19—H19C	109.5
C9—C8—C7	121.7 (2)	Al1—C20—H20A	109.5
C13—C8—C7	122.7 (2)	Al1—C20—H20B	109.5
F1—C9—C10	118.2 (3)	H20A—C20—H20B	109.5
F1—C9—C8	118.7 (3)	Al1—C20—H20C	109.5
C10—C9—C8	123.1 (3)	H20A—C20—H20C	109.5
C11—C10—C9	119.0 (3)	H20B—C20—H20C	109.5
C11—C10—H10	120.5	C1—N1—C14	109.6 (2)
C9—C10—H10	120.5	C1—N1—C15	112.6 (2)
C10—C11—C12	120.5 (3)	C14—N1—C15	105.8 (3)
C10—C11—H11	119.8	C1—N1—Al2	108.64 (17)
C12—C11—H11	119.8	C14—N1—Al2	117.10 (19)
C11—C12—C13	119.7 (3)	C15—N1—Al2	103.0 (2)
C11—C12—H12	120.1	C7—O1—Al2	122.10 (15)
C13—C12—H12	120.1	C7—O1—Al1	114.61 (15)
C12—C13—C8	122.2 (3)	Al2—O1—Al1	121.66 (10)
C12—C13—H13	118.9		
